# Rapidly Fatal Postlaparoscopic Liver Infection from the Rarely Isolated Species *Clostridium butyricum*

**DOI:** 10.1155/2020/1839456

**Published:** 2020-07-29

**Authors:** Kenneth L. Muldrew

**Affiliations:** Department of Pathology and Immunology, Baylor College of Medicine, Houston, TX, USA

## Abstract

We report a case of a rapidly fatal postlaparoscopic cholecystectomy liver infection from the rarely isolated species *Clostridium butyricum*. Liver examination at autopsy showed cystic spaces, necrosis, and spore-forming Gram-positive rods. 16sRNA gene sequencing of the cystic liver tissue identified the organism as *C. butyricum*.

## 1. Case Report

An adult patient underwent a laparoscopic cholecystectomy for symptomatic cholelithiasis. According to the operative report, the gallbladder was constricted and had minimal adhesions, a single gall stone, and a relatively short cystic duct. The gallbladder exhibited scarring and was removed. After multiple attempts, an intraoperative cholangiogram was aborted secondary to problems maintaining a seal and contrast leakage. The patient was discharged home, 6 hours after surgery.

During the first night at home after surgery, the patient was inactive, resting on the sofa, and slept uneventfully. The day after surgery, she spiked a 101°F temperature and called the hospital. The patient reportedly was told to take an over-the-counter fever reducer and call back if the fever did not abate. The patient remained ill throughout the day and was found dead the following day. Past medical history was significant for schizophrenia, glucose intolerance, and obesity, and prescribed medications included divalproex sodium, olanzapine, and hydrocodone. At autopsy, the decedent was 66 inches in length and weighed 257 pounds (BMI 42.8).

Three small surgical incisions (0.5 × 0.1 cm each) were apparent to the right of the midline approximately 2.0 cm from the umbilicus, and a 2.0 × 0.1 cm surgical incision overlying the umbilicus was also noted. All surgical incisions appeared to be clean and healing. The patient had cardiomegaly (560 grams) with slightly thickened mitral valve leaflets and no evidence of atherosclerotic heart disease. The liver weighed 1990 grams, and the surface demonstrated a greasy, yellow parenchyma consistent with hepatic steatosis. The gallbladder bed was normal for the postoperative period.

**Figure 1:**
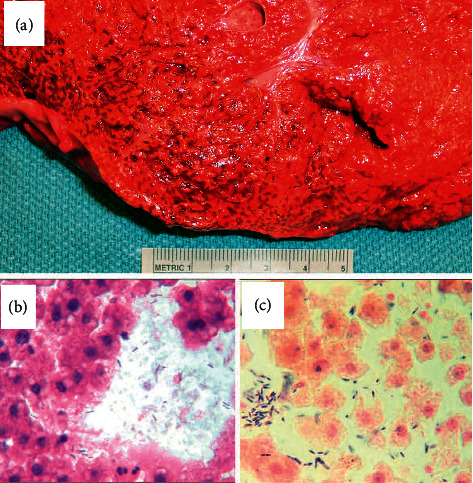
(a) Liver parenchyma at autopsy. (b) H&E stained section of the involved liver tissue (600x magnification). (c) Gram stain section of the involved liver tissue illustrating spore-forming Gram-negative rods (600x magnification).

Upon serial sectioning of the liver, 10.0 × 6.0 × 4.0 cm of small (0.1 cm) cystic spaces were located in the inferior portion of the liver; the consistency of the liver in this area was readily compressible, reminiscent of lung parenchyma (Figure 1(a)). Microscopic examination of the liver parenchyma demonstrated focal areas of necrosis, rare areas of acute inflammatory cells, vacuolated hepatocytes, and tissue air spaces (Figure 1(b); H&E section). On tissue Gram stain, copious amounts of Gram-positive rods with spores were present (Figure 1(c)). Toxicology results indicated the presence of diphenhydramine (too low to quantify) and bupivicaine. A postmortem liver swab sample was submitted for culture and demonstrated a rare diptheroid bacilli, rare Group F beta hemolytic Streptococcus, and rare Gram-positive bacilli, which the microbiology laboratory was unable to identify. Standard biochemical profile testing was used for identification of these organisms. At this time, evidence pointed to sepsis as the cause of death. Bacterial identification by 16s rRNA gene sequencing of a portion of the liver tissue was performed. As previously described, DNA extracted from the paraffin-embedded liver tissue was subjected to PCR amplification and sequencing of the first 500 base pairs of the bacterial 16S rRNA gene to identify the Gram-positive rod seen in the tissue sections [1, 2]. Comparison of the bacterial 16S rRNA gene sequence (GenBank accession number EU239262.1) obtained from the paraffin-embedded liver tissue to the MicroSeq bacterial 500 sequence database library (Applied Biosystems, Foster City, CA) and GenBank NCBI sequence database using the BLASTN algorithm [3] revealed 100.0% sequence similarity (no mismatches) with *C. butyricum*.

## 2. Discussion

*C. butyricum* is an anaerobic Gram-positive rod that appears as very large, irregular, mottled colonies on anaerobic blood agar. Microscopic examination details large subterminal endospores, and biochemical analysis demonstrates fermentation of many types of carbohydrates [4]. It is found both in the environment (as a soil bacterium) and in fecal isolates from humans and animals. *C. butyricum* is rarely reported as a human pathogen, but it has been implicated in various types of infections, including necrotizing enterocolitis [5– 8], polymicrobial peritonitis [9], type E botulism [10, 11], and several cases of *C. butyricum* bacteremia [12, 13]. *C. butyricum* sepsis occurred in one patient that injected cocaine into his indwelling central venous catheter [14].

Some strains of *C. butyricum* can produce type E botulinum neurotoxin, a potent protein toxin that has been linked to food-borne illnesses [15]. Although type E neurotoxin has been reported to be less severe and fatal than type A, the symptoms and mechanism of action are the same [16]. The mechanism of action of botulinum toxin begins with the binding of the toxin to synaptic vesicles of cholinergic nerves, which prevents the release of acetylcholine at the peripheral nerve endings, resulting in an acute, flaccid paralysis. The release of this toxin usually first presents in patients as vague gastrointestinal symptoms (abdominal cramps, nausea, vomiting, diarrhea, and constipation), which precede neurologic signs of descending flaccid paralysis within 24–48 hours. The paralysis with bilateral impairment of the cranial nerves then proceeds to paralysis of the face and finally descends to the thorax and extremities [4]. Death may occur after paralysis of the tongue or of the muscles of the pharynx leading to upper airway occlusion or by paralysis of the diaphragm and intercostal muscles. Importantly, the patient usually does not lose consciousness and remains cognizant of the symptoms [17].

Strains of *C. butyricum* that produce the type E neurotoxin have been linked to cases of infantile botulism, as well as botulism classified as adult infectious botulism or adult intestinal botulism. Adult intestinal botulism can occur in adults after abdominal surgery, in patients with gastrointestinal tract abnormalities, or in patients with recent antibiotic treatment that may have disrupted the natural intestinal flora [4, 18]. Some case reports of intestinal botulism describe patients presenting with acute abdominal pain that was suspected to be appendicitis. Two patients in one case review underwent appendectomies, revealing Meckel's diverticula, ascites, and intestinal loops distended by liquid and gas. The neurological symptoms typical of botulism evolved after surgery, and the patients were confirmed to have type E neurotoxin producing *C. butyricum* [15]. This patient did not have any intestinal symptoms premortem and had no lesions or other colonic disease at autopsy, which ruled out the intestinal form of infection.

The present case report describes a fatal case of postoperative sepsis due to *C. butyricum*. The incidence of surgical abdominal sepsis is dependent on the type of abdominal operation, the characteristics of the patient, and experience of the surgeon. It is estimated to be <2-3% for laparoscopic cholecystectomy. Patients who are aged, obese, or diabetic have higher a higher incidence of sepsis [19]. The decedent's obesity (257 lbs, BMI 42.8) and prediabetic state placed her in a subgroup of patients that have an increased risk of infection with the laparoscopic cholecystectomy procedure, but this does not completely explain how she was infected with *C. butyricum*. The surgeon did not recall anything out of the ordinary, and the sterile surgical field was uncompromised.

In our patient, along with sepsis, neurogenic toxin type E production may have contributed to her demise, but because of the rapidity of decline and death, wound botulism does not appear to be the main cause of death. Characteristically, the incubation period for wound botulism is 4–14 days [20]. Unfortunately, premortem serum had been destroyed by the hospital before neurogenic toxin type E assays could be performed. Next Generation Sequencing (NGS) has been used to identify toxin genes and drug resistance markers, but colony material was not isolated in culture so this technique could not be used in this case.

The possible sources of the infection include the gallbladder itself or an environmental contaminant during the surgical procedure. Operative notes describe a somewhat abnormal anatomy, including a short cystic duct and scarring of the gallbladder at the angle of Calot. However, the gallbladder did not appear gangrenous to either the surgeon or the pathologist, and therefore, the definitive source of the infection remains unknown.

The Emerging Infections Unit of the Minnesota Department of Health conducted an investigation and did not uncover any sign of hospital or community outbreak of *Clostridium butyricum* infection. Further investigation into the prevalence of this bacterium in both the hospital and community may be warranted.
